# Generalized yellow-white papules in a patient with acute joint pain

**DOI:** 10.1016/j.jdcr.2025.05.034

**Published:** 2025-06-19

**Authors:** Ahmed M. Eldaboush, Travis Vandergriff, Arturo R. Dominguez

**Affiliations:** a Department of Dermatology, Perelman Schools of Medicine, University of Pennsylvania, Philadelphia, Pennsylvania; b Department of Dermatology, University of Texas Southwestern Medical Center, Dallas, Texas; c Department of Internal Medicine, University of Texas Southwestern Medical Center, Dallas, Texas

**Keywords:** cutaneous deposits

A 46-year-old male with a history of obesity and prediabetes presented with a 3-day history of left wrist and elbow pain and swelling along with a 6-month history of intermittent left knee pain that had recently worsened. He had also reported painless abdominal bumps and nodules for 8 months, which expressed a white material (Figs 1 and 2). Examination demonstrated yellow to white milia-like papules and nodules distributed across the abdomen, flanks, chest, arms, and legs. Several lesions on the abdomen were exophytic with central extrusion of chalky white material, accompanied by surrounding pink to red erythema. A subset of these lesions also exhibited ulceration with overlying crust (Fig 1). His left knee, bilateral elbows, left wrist, and first carpometacarpal joints were erythematous, swollen, and tender with reduced motion. His vital signs were within normal limits. Laboratory results showed normal renal function with a hemoglobin A1c of 5.9%.

**Fig 1:**
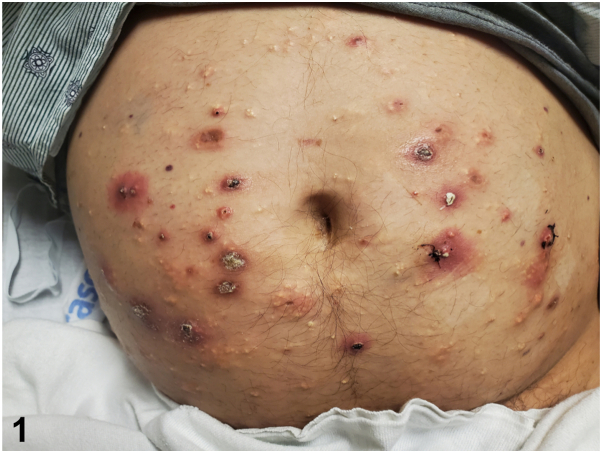

**Fig 2:**
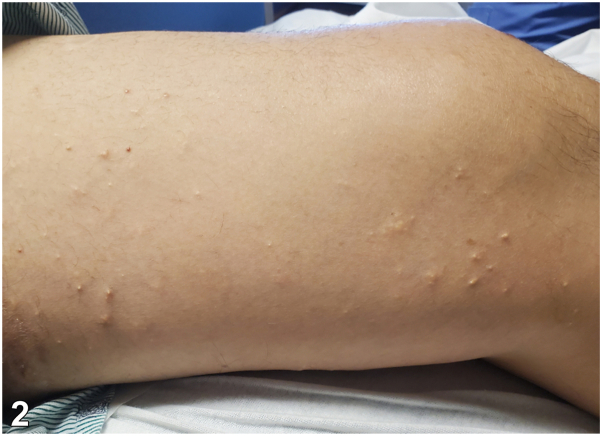

**Question 1: What is the best next step in evaluating and managing this patient?**

A. Order antinuclear antibodies
B. Recommend arthrocentesis with synovial fluid analysis
C. Order serum fasting triglycerides
D. Measure serum calcium and phosphate
E. Measure serum uric acid

**Answers:**

A. Order antinuclear antibodies – Incorrect. Connective tissue diseases such as systemic sclerosis and dermatomyositis can present with calcinosis cutis in adults, especially in longstanding cases. However, other suggestive features of connective tissue diseases were absent in this patient.
B. Recommend arthrocentesis with synovial fluid analysis – Correct. Gouty tophi result from the deposition of monosodium urate crystals within soft tissues, most commonly in periarticular regions.^1^ They typically develop in longstanding gout, usually after about 10 years of recurrent polyarticular flares, and rarely present as the initial sign of disease.^2^ Miliarial tophi are a rare intradermal form of tophaceous gout that presents as small, white, milia-like papules filled with chalky material, as seen in this patient. Two-thirds of cases have occurred in patients without a prior diagnosis of gout and represented the first sign of tophaceous disease.^3^^,^^4^ Miliarial tophi often involve nonperiarticular skin, especially the thighs and shins,^4^ unlike classic tophi which have a predilection to periarticular skin. Treatment with urate-lowering medications such as allopurinol or febuxostat has shown variable results. Among 11 published cases, 1 case showed complete resolution after 3 months of therapy, 4 cases showed partial improvement over an average of 8 and a half months, and 6 cases were lost to follow-up or had no outcome reported.^4^ Given the atypical presentation and lack of gout history, both arthrocentesis and skin biopsy are indicated to confirm the diagnosis in this patient.
C. Order serum fasting triglycerides – Incorrect. Eruptive xanthomas are small, pruritic yellow-to-orange papules, sometimes with surrounding erythema. Eruptive xanthomas are associated with severe hypertriglyceridemia, and not arthritis.
D. Measure serum calcium and phosphate – Incorrect. Secondary, sporadic tumoral calcinosis is characterized by dermal calcium phosphate deposits, typically around large joints, and is usually associated with secondary hyperparathyroidism in the setting of end-stage renal disease. Lesions can grow large (up to 20 cm), causing disfigurement and impaired function. Calcium-phosphorus product may be elevated.
E. Measure serum uric acid – Incorrect. While elevated uric acid levels are associated with gout, they are neither diagnostic nor specific. Many individuals with hyperuricemia do not develop gout, and uric acid levels can be normal during an acute flare.

**Question 2: Which of the following is not known to be a risk factor for this condition?**

A. Obesity
B. Diuretic use
C. Chronic kidney disease
D. Vitamin C supplementation
E. Excessive alcohol consumption

**Answers:**

A. Obesity – Incorrect. Obesity, along with other components of metabolic syndrome such as hypertension and dyslipidemia, is a well-established risk factor for gout.^5^ Body mass index >30 kg/m^2^ was associated with a 2-fold increased risk of developing gout.^6^
B. Diuretic use – Incorrect. Diuretics, particularly loop and thiazide diuretics, are associated with an approximately 2-fold increased risk of developing gout.^5^ This is mainly due to volume depletion, which enhances uric acid reabsorption in the proximal tubule. Some diuretics may also directly impair uric acid excretion, while a few can have a mild uricosuric effect.^5^ The risk is higher with loop diuretics than with thiazides and tends to be more pronounced in men.^5^ Additionally, underlying conditions treated with diuretics, such as hypertension, are themselves linked to increased gout risk.^5^
C. Chronic kidney disease – Incorrect. Chronic kidney disease is an established independent risk factor for gout^7^ as well as for the incidence of miliarial tophi.^4^
D. Vitamin C supplementation – Correct. Although high doses of vitamin C (>1000 mg/day) may increase the risk of uric acid kidney stones in some individuals, it is not a known risk factor for gout. In fact, vitamin C supplementation can lower serum uric acid levels^8^ by enhancing renal excretion. However, current observational and interventional studies do not support its use for preventing the onset or recurrence of gout.
E. Excessive alcohol consumption – Incorrect. Excessive alcohol consumption is associated with a 2-fold higher risk of developing gouty tophi.^5^

**Question 3: What are the characteristic histologic features seen in a skin biopsy of this lesion?**

A. Irregular deeply basophilic deposits of amorphous material within the dermis and subcutaneous tissue.
B. Deposits of amorphous, feathery material and needle-shaped crystals within the dermis and subcutis surrounded by a granulomatous infiltrate with multinucleated giant cells.
C. A dense infiltrate of lipid-laden foamy histiocytes with a mixed lymphocytic and neutrophilic infiltrate and free extracellular lipid.
D. Dense eosinophilic deposits within the dermis with spicules of bone perforating the overlying epidermis.
E. Noncaseating granuloma with scarce lymphocytic inflammation surrounded by a rim of dermal fibrosis.

**Answers:**

A. Irregular deeply basophilic deposits of amorphous material within the dermis and subcutaneous tissue – Incorrect. This describes the histopathology of calcinosis cutis.
B. Deposits of amorphous, feathery material and needle-shaped crystals within the dermis and subcutis surrounded by a granulomatous infiltrate with multinucleated giant cells – Correct. This describes the characteristic histopathology of gouty tophi, as observed in this patient's lesions (Fig 3).

**Fig 3:**
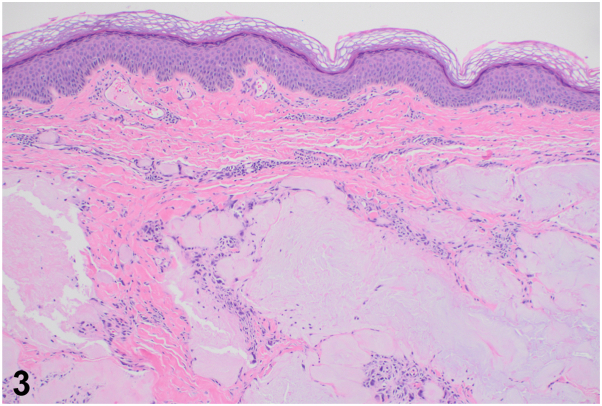
C. A dense infiltrate of lipid-laden foamy histiocytes with a mixed lymphocytic and neutrophilic infiltrate and free extracellular lipid – Incorrect. This describes the histopathology of eruptive xanthomas.
D. Dense eosinophilic deposits within the dermis with spicules of bone perforating the overlying epidermis – Incorrect. This describes the histopathology of perforating osteoma cutis.
E. Noncaseating granuloma with scarce lymphocytic inflammation surrounded by a rim of dermal fibrosis – Incorrect. This describes the histopathology of sarcoidosis.

## Conflicts of interest

None disclosed.
